# The glycoprotein GPNMB protects against oxidative stress through enhanced PI3K/AKT signaling in epidermal keratinocytes

**DOI:** 10.1016/j.jbc.2025.108299

**Published:** 2025-02-11

**Authors:** Natsuki Nishida, Mariko Otsu, Yukiko Mizutani, Asako Ishitsuka, Yoichi Mizukami, Shintaro Inoue

**Affiliations:** 1 Department of Cosmetic Health Science, Gifu Pharmaceutical University, Gifu, Japan; 2 Center for Gene Research, Yamaguchi University, Yamaguchi, Japan

**Keywords:** vitiligo, GPNMB, human epidermal keratinocytes, oxidative stress, PI3K/AKT, WNT/β-catenin

## Abstract

Vitiligo, an autoimmune disease caused by environmental and genetic factors, is characterized by the specific loss of epidermal melanocytes (MCs). IFN-**γ**, predominantly derived from MC-targeting CD8^+^ T cells, plays a key role in vitiligo pathogenesis. Previously, we found that glycoprotein nonmetastatic melanoma protein B (GPNMB) is specifically lost in the basal epidermal layer of vitiligo lesions and downregulated by IFN-**γ** in normal human epidermal keratinocytes (KCs) (NHEKs). This study aimed to determine the role of KC GPNMB in normal and vitiligo epidermis and demonstrated that GPNMB plays a protective role against H_2_O_2_-induced oxidative stress due to its extracellular domain. In contrast, the NRF2/KEEP1 system was not involved in the anti-oxidative response in NHEKs but was active in MCs. GPNMB knockdown reduced the phosphorylation levels of AKT^T308^ and AKT^S473^ after H_2_O_2_ treatment, accompanied by reduced Dickkopf-1 (DKK1) mRNA and protein production and decreased FOXM1 mRNA expression. These results suggested that GPNMB protects KCs from H_2_O_2_-induced cell death through enhanced PI3K/AKT signaling, and WNT/**β**-catenin/FOXM1 and DKK1/CKAP4/AKT pathways. Furthermore, a significant increase in thioredoxin-interacting protein (TXNIP) following GPNMB knockdown was observed, indicating the enhanced phosphorylation of JNK and p38 and suppression of WNT/**β**-catenin signaling. These results suggest that the decreased expression of epidermal GPNMB in vitiligo lesions triggers increased sensitivity to H_2_O_2_-induced oxidative stress and decreased WNT/**β**-catenin signaling, consistent with the pathological features of the vitiligo epidermis. These findings may enhance our understanding of vitiligo pathogenesis, provide insights into the reduced risk of epidermal cancers, and highlight novel targets for treatment.

Vitiligo is a complex multifactorial autoimmune disease induced by environmental, genetic, and immune factors and is characterized by the specific loss of epidermal melanocytes (MCs). MCs are damaged by pre-exposure to environmental factors, such as oxidative stress, and display MC-specific antigens (1, 2, 3, 4). Consequently, MCs are persistently eliminated from the epidermis through the action of IFN-γ, granzyme B, and perforin, which are primarily produced by MC-targeting CD8^+^ T cells (5, 6). However, recent studies have shown that non-acquired immune cells, such as innate immune cells (natural killer cells and Type 1 innate lymphoid cells) (7, 8), keratinocytes (KCs) (9, 10), fibroblasts (11, 12), and mast cells (13, 14), are involved in the development and/or maintenance of vitiligo pathogenesis. Increased H_2_O_2_ levels have been detected in the epidermis of patients with vitiligo (15), along with decreased levels of catalase and increased levels of superoxide dismutase (SOD) (16). Thus, when these cells are exposed to oxidative stress, primarily induced by H_2_O_2_, they may eliminate MCs directly or indirectly through non-immune mechanisms, including detaching MCs from the basement membrane (17, 18) and reactive oxygen species (ROS)-induced (19, 20) or IFN-γ-dependent MC death and senescence (21, 22). In particular, vitiligo MCs are well known to be vulnerable to oxidative stress because of the impaired activation of NF-E2-related factor 2 (NRF2) under oxidative stress (23).

In addition, both MCs and KCs are damaged in vitiligo lesions and exhibit weak E-cadherin expression and an increase in the number of apoptotic caspase-3-positive cells; these lesions are also characterized by hyperkeratosis, basal vacuolization, acanthosis, ballooning, and spongiosis (9). Furthermore, apoptosis-induced KCs decrease stem cell factor (SCF) production, leading to a decrease in MCs (24). Although there have been several reports on the anti-oxidative stress activity of NRF2 in cultured human KCs, most evidence has been obtained using HaCaT cells, an immortalized human KC strain that differs from primary normal human epidermal KCs (NHEKs) (25, 26). Thus, it remains unclear whether vitiligo KCs are more sensitive to ROS, in a manner similar to MCs, and whether NRF2 is responsible for the antioxidant capacity of KCs.

Our previous study revealed that the expression of glycoprotein nonmetastatic melanoma protein B (GPNMB, alias osteoactivin/dendritic cell-associated transmembrane protein) is specifically lost in the basal epidermal layer of vitiligo lesions, but not in depigmented lesions in the tuberous sclerosis complex (27). Moreover, GPNMB is substantially expressed in cultured NHEKs under low Ca^2+^ conditions (0.07 mM) and, notably, downregulated by IFN-γ, which plays a critical role in vitiligo onset and maintenance (27).

GPNMB is a type-I transmembrane protein that is expressed not only in cancer cells, but also in many normal cells, including KCs (27), MCs (28, 29), macrophages (30, 31), dendritic cells (32, 33), osteoblasts (34, 35), microglia (36, 37), and neurons (38, 39). GPNMB has been associated with various pathophysiological events, including cell adhesion (40, 41), differentiation (42), senescence (43), inflammatory responses (30, 44, 45), neuronal degeneration (46, 47), bone formation (35, 42), T-cell activation (48, 49), and metastasis in several cancers (49, 50, 51). The extracellular domain of GPNMB is cleaved by a disintegrin and metalloprotease 10 (AMDM10) to produce soluble GPNMB (sGPNMB) (27, 52). This form contains an RGD motif that binds to integrins to facilitate cell-cell adhesion, and an Ig-like polycystic kidney disease domain involved in protein-protein and protein-carbohydrate interactions. The intracellular domain contains an immunoreceptor tyrosine-based activation motif for intracellular signaling *via* Src and Syk cytoplasmic kinases and a di-leucine motif for endosomal and melanosomal sorting (53). sGPNMB interacts with various receptors, including Na^+^/K^+^-ATPase (46, 54), CD44 (31, 44), integrins (40), and syndecan-4 (48, 55). The glioma progression and neuroprotective effects of sGPNMB are mediated *via* the activation of the phosphoinositide 3-kinase (PI3K)/AKT and mitogen-activated protein kinase (MAPK)-extracellular signal-regulated kinase (ERK) kinase (MEK)/ERK pathways *via* binding to the α-subunit of Na^+^/K^+^-ATPase (46, 54). Moreover, human sGPNMB protects MCs from oxidative stress in a CD44-independent manner (56), while mouse sGPNMB exhibits neuroprotective properties by attenuating astrocyte-mediated neuroinflammation in a CD44-dependent manner (46). However, the precise physiological function of GPNMB in NHEKs, and its role in vitiligo, remain unclear.

Therefore, we focused on the sensitivity of KCs to H_2_O_2_-induced oxidative stress in vitiligo lesions with high epidermal H_2_O_2_ levels accompanied by reduced catalase and increased SOD activity (15, 16). First, we investigated the intracellular signaling changes after H_2_O_2_-induced oxidative stress in cultured NHEKs. Then, GPNMB was knocked down to mimic vitiligo KCs to investigate the role of GPNMB and its underlying mechanisms in cell susceptibility to H_2_O_2_ treatment. The findings clarify the role of GPNMB in KCs and provide novel insights into the pathophysiology of vitiligo for clinical applications.

## Results

### Knocking down of GPNMB, but not NRF2, increases H_2_O_2_-induced cytotoxicity in NHEKs

**Figure 1:**
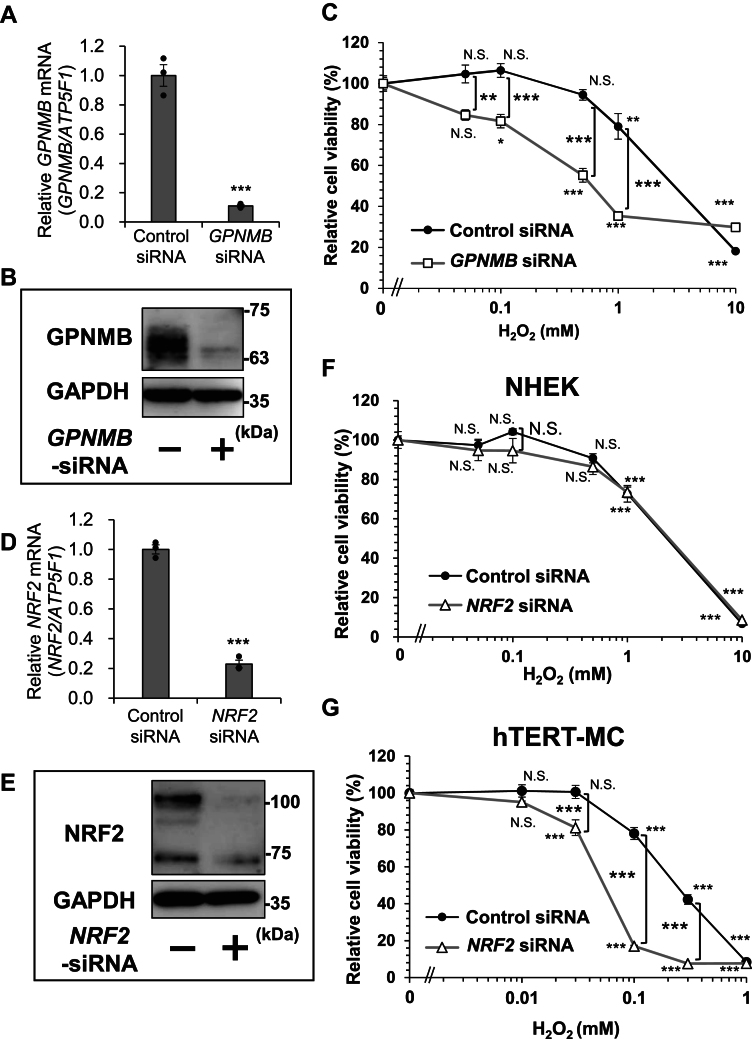
**Knocking down GPNMB, but not NRF2, increases H**_**2**_**O**_**2**_**-induced cytotoxicity in normal human epidermal keratinocytes (NHEKs).***A–C*, following transfection with *GPNMB* siRNA, NHEKs were cultured in basal medium, and samples were collected after 48 h (*A*) or 72 h (*B*). GPNMB expression levels in NHEKs were measured by qRT-PCR (*A*) or immunoblotting (*B*). *C*, the cytotoxicity of H_2_O_2_ was detected using a Cell Counting Kit-8. After transfection of control (*closed circle*) or *GPNMB* (*open square*) siRNA for 48 h, NHEKs were stimulated with H_2_O_2_ (0.03–10 mM) for 24 h. *D–F*, after transfection of *NRF2* siRNA, NHEKs were cultured in basal medium and samples were collected after 48 h (*D*) or 72 h (*E*). NRF2 expression levels in NHEKs were measured by qRT-PCR (*D*) or immunoblotting (*E*). *F*, the cytotoxicity of H_2_O_2_ was detected using a Cell Counting Kit-8. After transfection of control (*closed circle*) or *NRF2* (*open triangle*) siRNA for 48 h, NHEKs were stimulated with H_2_O_2_ (0.03–10 mM) for 24 h. *G*, in hTERT MCs, the cytotoxicity of H_2_O_2_ was measured by a Cell Counting Kit-8. After transfection of control (*closed circle*) or *NRF2* (*open triangle*) siRNA for 48 h, h-TERT MCs were stimulated with H_2_O_2_ (0.01–1.0 mM) for 24 h. The loading controls were *ATP5F1* (for RT-qPCR) and GAPDH (for immunoblotting). The expression levels in *GPNMB* siRNA-treated cells are shown as a ratio, and the expression of each target in control siRNA-treated cells was set at 1.00. Values represent the mean ± S.E.M. (*A*, *D*, and *G*: n = 3, C: n = 6, F: n = 8). ∗*p* < 0.05, ∗∗*p* < 0.01, ∗∗∗*p* < 0.001 (Student's *t* test [*A* and *D*] or Tukey's test [*C*, *F*, and *G*]). N.S., not significant.

A dose-dependent decrease in the cell viability was observed when NHEKs were treated with H_2_O_2_ (0.03–10 mM, IC_50_ = 3 mM), which was enhanced by knocking down GPNMB using a specific small-interfering RNA (siRNA) (IC_50_ = 0.6 mM) (Fig. 1, *AC*). Conversely, the viability curve remained unchanged after knocking down NRF2 (Fig. 1, *DF*). While NRF2 knockdown in human immortalized MCs enhanced H_2_O_2_ (0.01–1.0 mM) -induced cytotoxicity, reducing the IC_50_ to 0.054 mM from 0.24 mM (Fig. 1*G*). GPNMB knockdown demonstrated similar effects in NHEKs derived from a different donor (Fig. S1*A*). These results indicated that GPNMB is involved in cytoprotection against oxidative stress-induced damage in NHEKs, whereas NRF2 exerts protective effects in human MCs but not in NHEKs.

**Figure 2:**
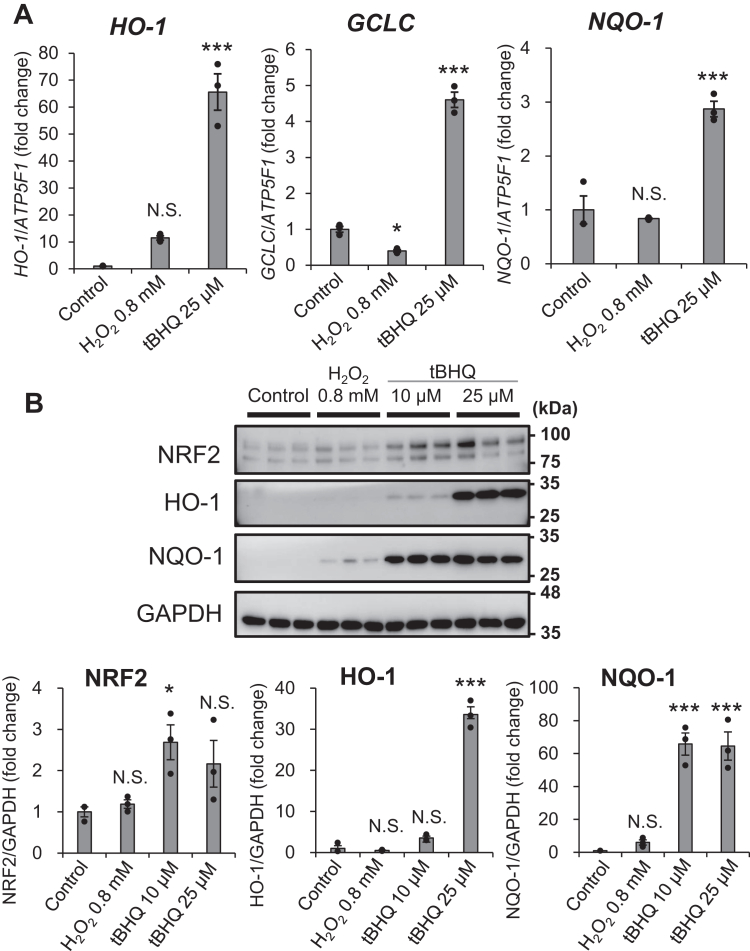
**NRF2 signaling does not function as a sensor for H**_**2**_**O**_**2**_**-induced oxidative stress in NHEKs**. *A*, in NHEKs, the mRNA expression levels of *HO*-*1*, *GCLC*, and *NQO-1* mRNA were detected by qRT-PCR. Following treatment with or without H_2_O_2_ (0.8 mM) and *t*BHQ (25 μM) for 24 h, the total RNA of NHEKs was collected. Values represent the mean ± S.E.M. (n = 3). ∗*p* < 0.05, ∗∗*p* < 0.01, ∗∗∗*p* < 0.001 *versus* the non-treated control (Tukey's test). Expression levels were normalized to *ATP5F1* as a loading control. *B*, expression of NRF2, HO-1, and NQO-1 proteins was detected by immunoblotting. NHEKs were treated with H_2_O_2_ (0.8 mM) or tBHQ (10 or 25 μM) for 24 h. Glyceraldehyde 3-phosphate dehydrogenase (GAPDH) was used as the immunoblotting loading control. Relative band intensity was calculated from the immunoblotting data. The non-treated control was set at 1.0, and the fold change is shown as a ratio. Values represent the mean ± S.E.M. (n = 3). ∗*p* < 0.05, ∗∗*p* < 0.01, ∗∗∗*p* < 0.001 *versus* the non-treated control (Tukey's test). N.S., not significant.

To confirm these results (Fig. 1*F*), we examined whether NHEKs express NRF2-targeting genes such as *heme oxygenase 1 (HO-1), glutamate–cysteine ligase catalytic subunit (GCLC),* and *NAD(P)H quinone dehydrogenase 1 (NQO-1)* under H_2_O_2_-induced oxidative stress. Treatment with tertiary butylhydroquinone (*t*BHQ, 25 μM), an NRF2 activator (57), significantly induced the mRNA expression of these three genes (*p* < 0.001). In contrast, H_2_O_2_ treatment (0.8 mM) downregulated *GCLC* mRNA (*p* < 0.05) expression, while *NQO-1* and *HO-1* mRNA expression remained unchanged (Fig. 2*A*). Immunoblotting analyses revealed activated NRF2, HO-1, and NQO-1 protein expression 24 h after the addition of *t*BHQ (10 and 25 μM); however, no significant levels of these antioxidant proteins were measured in control and H_2_O_2_-treated NHEKs (Fig. 2*B*). Cells pretreated with the proteasome inhibitor MG132, which stabilizes the NRF2-KEEP1 complex, confirmed NRF2 protein expression in both control and H_2_O_2_-stimulated cells. However, HO-1 and NQO-1 protein expression was not induced by H_2_O_2_ (Fig. S2). This indicates that NHEKs have a functional NRF2 antioxidant system responsible for activating NRF2; however, the NRF2-KEEP1 system in NHEKs may not function as a sensor for H_2_O_2_-induced oxidative stress. Given that GPNMB has been shown to play an essential role in the anti-oxidative response, the effects of GPNMB knockdown on changes in the signal transduction response of NHEKs were examined after treatment with H_2_O_2_.

### GPNMB knockdown suppresses H_2_O_2_-induced AKT phosphorylation while enhancing p38 and ERK phosphorylation

**Figure 3:**
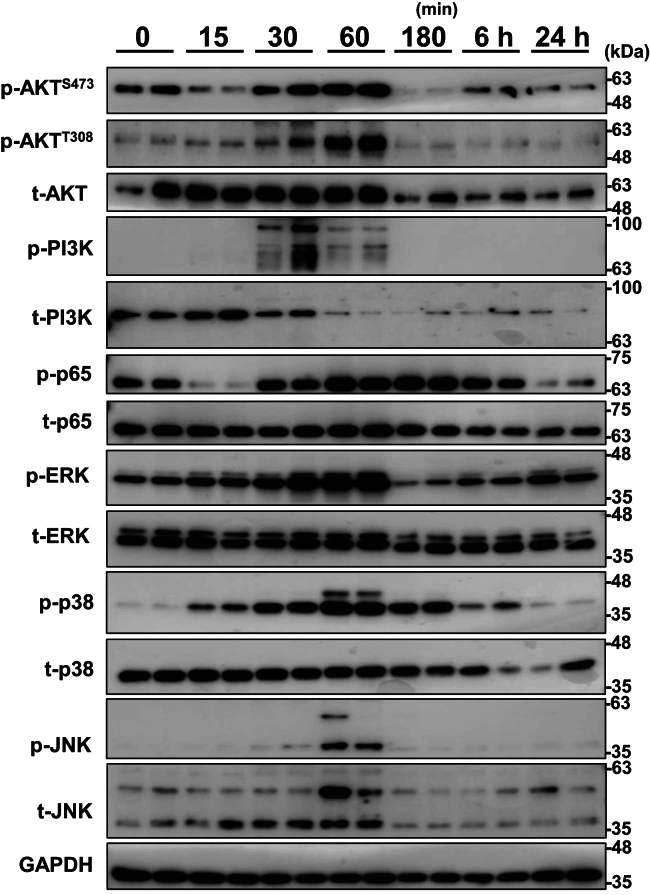
**H**_**2**_**O**_**2**_**induces time-dependent signal activation in NHEKs.** NHEK cells were treated with 0.8 mM of H_2_O_2_ for 15 min to 24 h. Phosphorylated and total AKT, PI3K, p65, ERK, p38, and JNK protein expression in cell lysates was analyzed by immunoblotting. GAPDH was used as a loading control and is displayed as representative data (Gel No. 6), which was obtained from twelve gel data for analyzing each protein in the same sample (Fig. S3). p-, phosphorylated; t-, total.

When NHEKs were treated with 0.8 mM of H_2_O_2_, which resulted in 80% survival after 24 h of incubation, AKT^S473^ and PI3K were phosphorylated within 30 min of H_2_O_2_ treatment, followed by AKT^T308^ phosphorylation at 60 min, all of which disappeared after 120 min (Fig. 3). ERK exhibited peak phosphorylation at 60 min, followed by sustained weak phosphorylation for up to 24 h. JNK was transiently phosphorylated at 60 min, and p38 was phosphorylated from 15 min to 6 h, reaching peak levels at 60 min. In addition, NF-κB p65 (RELA) was phosphorylated from 30 min to 6 h, reaching maximum levels between 60 and 180 min t-AKT, t-PI3K, and t-JNK expression levels markedly decreased upon phosphorylation (Fig. 3 and S3).

**Figure 4:**
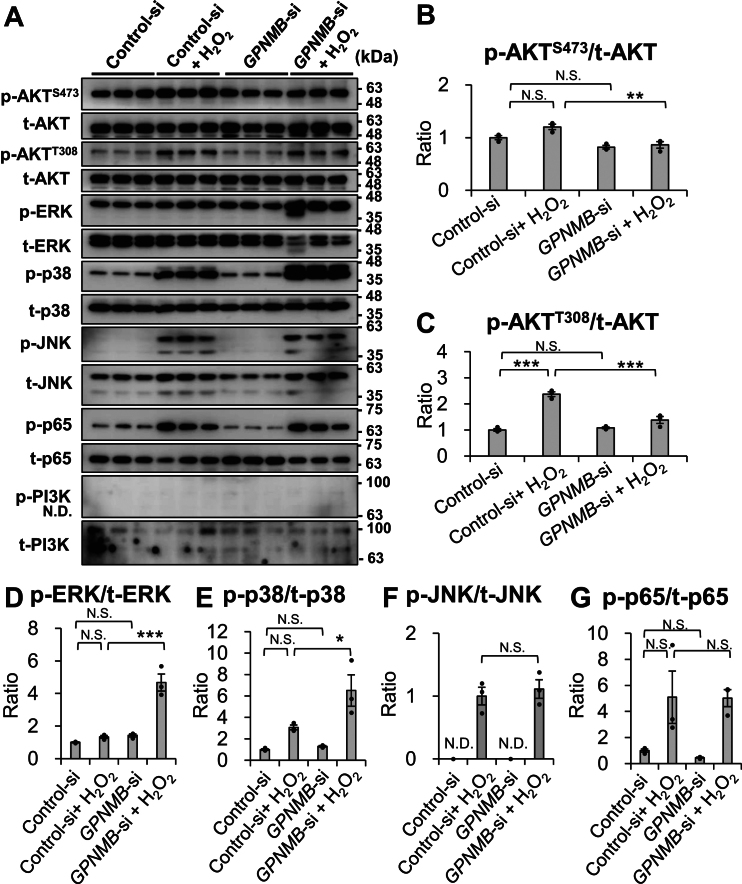
**GPNMB knockdown suppresses H**_**2**_**O**_**2**_**-induced AKT phosphorylation while enhancing p38 and ERK phosphorylation**. *A*, NHEKs were treated with or without *GPNMB* siRNA for 72 h and incubated with or without 0.8 mM H_2_O_2_ for 60 min. Phosphorylated and total AKT, ERK, p38, JNK, p65, and PI3K protein expression in cell lysates were analyzed by immunoblotting. *B–G*, relative band intensity was calculated, and phosphorylation was normalized to each total protein expression band. The control siRNA without H_2_O_2_ was set at 1.0, and fold change is presented as a ratio. Values represent the mean ± S.E.M. (n = 3). ∗*p* < 0.05, ∗∗*p* < 0.01, ∗∗∗*p* < 0.001 *versus* non-treated control (Tukey's test). p-: phosphorylated, t-: total. N.D., not detected; N.S., not significant.

Because ROS-induced cellular responses were observed in NHEKs treated with an 80% survival dose of H_2_O_2_, the effects of GPNMB knockdown on signal transduction were examined 60 min after H_2_O_2_ treatment (Fig. 4). GPNMB knockdown 48 h before H_2_O_2_ treatment suppressed the H_2_O_2_-induced phosphorylation of AKT at T308 and S473 (Fig. 4, *A*–*C*). In contrast, H_2_O_2_-induced phosphorylation of ERK and p38 was significantly increased by GPNMB knockdown (*p* < 0.001 and *p* < 0.05, respectively) (Fig. 4, *D* and *E*). However, H_2_O_2_-induced phosphorylation levels of JNK and p65 were not affected by GPNMB knockdown (Fig. 4, *F* and *G*). Moreover, phosphorylated PI3K was not detected at 60 min because it was transiently phosphorylated at 30 min and disappeared immediately (Fig. 4*A*). These results indicate that decreased GPNMB expression modulates ROS-induced AKT, ERK, and p38 phosphorylation to regulate cell survival and death.

### GPNMB knockdown attenuates Dickkopf-1 (DKK1) expression and secretion from NHEKs and suppresses the PI3K/AKT pathway

**Figure 5:**
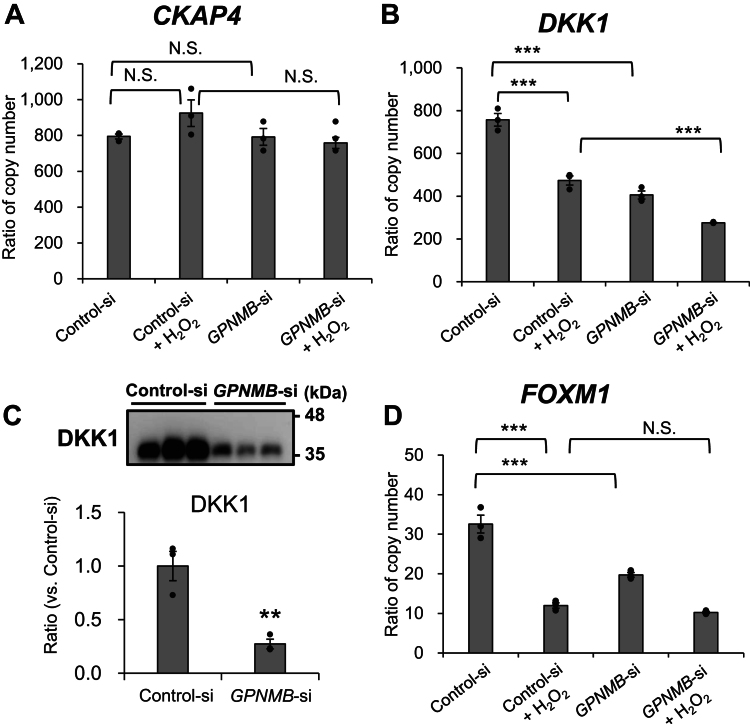
**GPNMB knockdown attenuates Dickkopf-1 (DKK1) expression and secretion from NHEKs and suppresses the PI3K/AKT signaling pathway.** NHEKs were transfected with NT or *GPNMB* siRNA for 72 h and incubated with or without 0.8 mM H_2_O_2_ for 24 h. The library was prepared from poly(A) mRNA using the NEBNext Ultra II RNA library prep kit and analyzed on a NextSeq 500. *A* and *B*, expression levels of *CKAP4* (DKK1 receptor) and *DKK1* mRNA in NHEKs were determined by next-generation sequencing. The TPM was calculated based on gene length and read count, and the relative expression level was corrected to the TPM of *ATP5F1*, a housekeeping gene. *C*, immunoblotting was performed to detect the levels of DKK1 protein released into the conditioned media of NHEKs. The expression level was calculated, and the expression of the control siRNA was set to 1.0. *D*, the relative expression level of *FOXM1* mRNA in NHEKs was determined by correcting the TPM calculated by next-generation sequencing with the TPM of *ATP5F1*. Values represent the mean ± S.E.M. (n = 3). ∗*p* < 0.05, ∗∗*p* < 0.01, ∗∗∗*p* < 0.001 (Tukey's test). TPM: transcripts per million. N.S., not significant.

To elucidate the mechanism through which decreased GPNMB expression suppresses AKT activation, we examined the cytoskeleton-associated protein 4 (CKAP4) receptor-mediated PI3K/AKT pathway (58, 59). Although the mRNA expression levels of *CKAP4* were not altered by H_2_O_2_ treatment or GPNMB knockdown (Fig. 5*A*), *DKK1* expression was significantly reduced by H_2_O_2_, GPNMB knockdown, and GPNMB knockdown + H_2_O_2_ treatment (*p* < 0.001) (Fig. 5*B*). Immunoblotting analysis showed that secreted DKK1 in the conditioned media decreased significantly following GPNMB knockdown (*p* < 0.01) (Fig. 5*C*). These results suggest that the DKK1/CKAP4/PI3K/AKT axis is downregulated, as indicated by the decreased AKT phosphorylation (Fig. 3, *A*–*C*). The mRNA levels of FOXM1, a transcription factor functioning downstream of DKK1/CKAP4/PI3K/AKT signaling (60), were significantly reduced by H_2_O_2_ treatment and GPNMB knockdown (*p* < 0.001) (Fig. 5*D*). This resulted in decreased DKK1 production by this pathway as well as through the WNT/β-catenin/FOXM1 pathway.

### The thioredoxin (TXN)/TXN-interacting protein (TXNIP) system is involved in increased p38 and ERK phosphorylation

**Figure 6:**
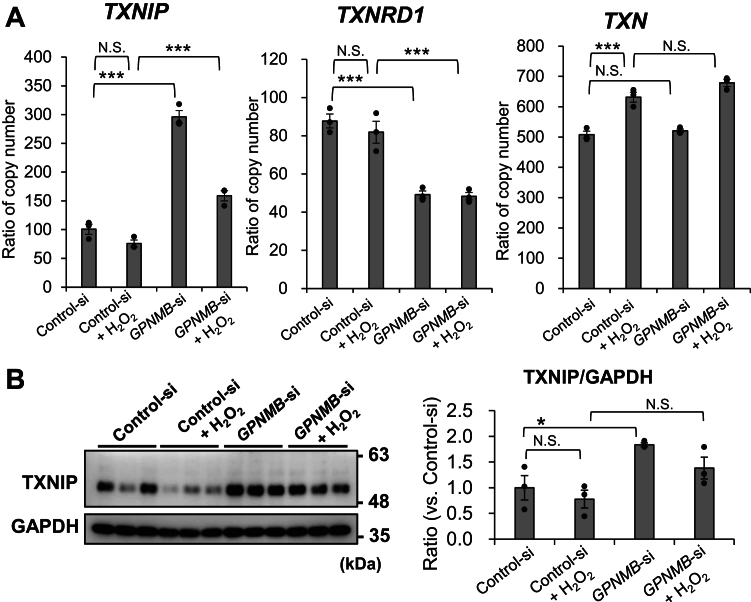
**Thioredoxin-interacting protein (TXNIP) expression is increased by GPNMB suppression**. NHEKs were treated with or without GPNMB siRNA for 72 h and then incubated with or without 0.8 mM H_2_O_2_ for 24 h. *A*, the relative expression levels of *TXNIP*, *thioredoxin reductase 1 (TXNRD1)*, and *TXN* mRNA in NHEKs were determined by correcting the TPMs calculated by next-generation sequencing with the TPM of *ATP5F1*. *B*, immunoblotting was performed to detect TXNIP expression in NHEKs. GAPDH was used as a loading control. The expression of the control siRNA was set to 1.0. Values represent the mean ± S.E.M. (n = 3). ∗*p* < 0.05, ∗∗*p* < 0.01, ∗∗∗*p* < 0.001 (Tukey's test). TPM: transcripts per million. N.S., not significant.

ROS-induced phosphorylation of ERK, p38, and JNK (Fig. 3) is mediated by both the RAS/RAF/MEK pathway and apoptosis signal-regulating kinase (ASK) (61). NHEKs primarily expressed *ASK2* mRNA (59.2 ± 0.8 transcripts/million (TPM)) as compared to *ASK1* mRNA (8.1 ± 0.6 TPM), and *ASK2* mRNA was upregulated by H_2_O_2_ treatment (81.9 ± 0.3 TPM). However, the expression levels of the ASK2 protein were not determined because the specific antibody was not available. Therefore, we examined the factors regulating ASK2 levels. TXNIP releases freely available ASKs from the inert TXN/ASK complex to form the TXN/TXNIP complex (61, 62). GPNMB knockdown significantly increased TXNIP at the mRNA and protein levels (*p* < 0.001 and *p* < 0.01, respectively) (Fig. 6, *A* and *B*). Moreover, GPNMB knockdown significantly decreased thioredoxin reductase 1 (*TXNRD1*) mRNA levels (*p* < 0.001) (Fig. 6*A*). *TXN* mRNA expression was upregulated by H_2_O_2_ treatment (*p* < 0.001) but was not affected by GPNMB knockdown.

### Recombinant ectodomain of GPNMB partially alleviates H_2_O_2_-induced NHEK cytotoxicity enhanced by GPNMB knockdown

**Figure 7:**
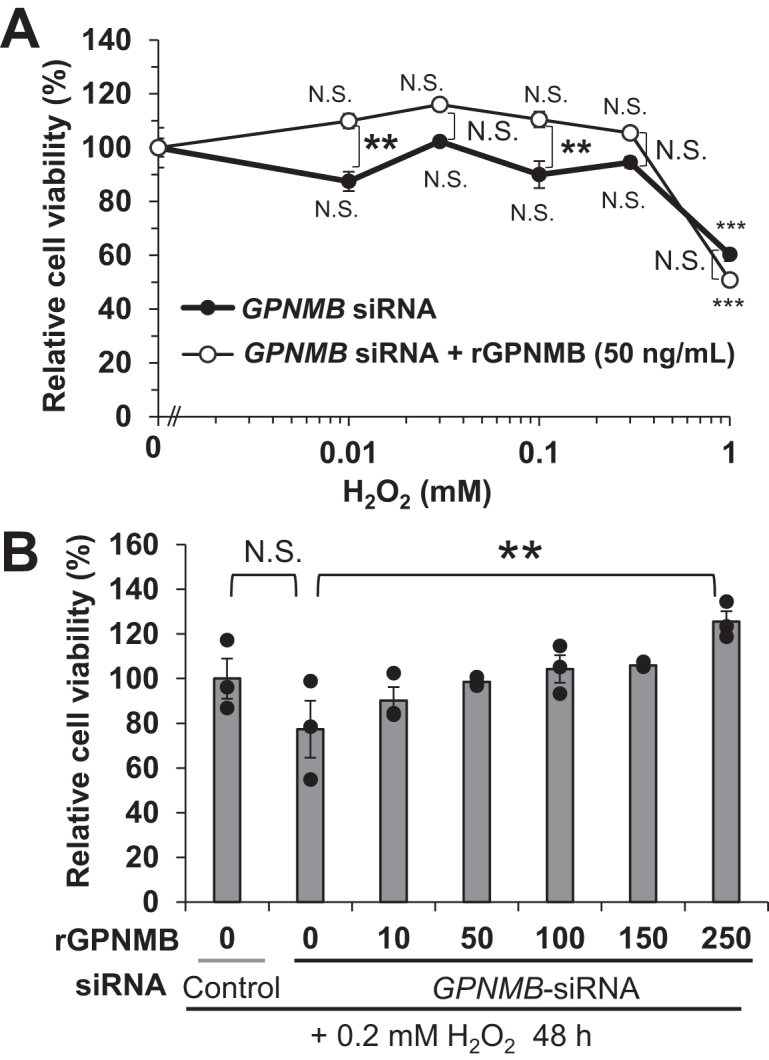
**rGPNMB alleviates H**_**2**_**O**_**2**_**-mediated cytotoxicity in NHEKs**. *A*, NHEKs were treated without *GPNMB* siRNA for 72 h. After knocking down GPNMB, cells were treated with 0.1 to 1.0 mM H_2_O_2_ with or without rGPNMB (50 ng/ml) for 24 h. *B*, dose-dependent reaction of rGPNMB. After transfection of *GPNMB* siRNA, NHEKs were cultured in basal medium for 48 h. After the downregulation of GPNMB, H_2_O_2_ (0.2 mM) was added in basal medium containing rGPNMB (10–250 ng/ml) for 48 h. The cytotoxicity of H_2_O_2_ was measured using Cell Counting Kit-8. Values represent the mean ± S.E.M. (*A*: n = 6, *B*: n = 3). ∗*p* < 0.05, ∗∗*p* < 0.01, ∗∗∗*p* < 0.001 (Tukey’s test). N.S., not significant.

The GPNMB extracellular domain (ectodomain) is released from cells as soluble GPNMB *via* ADAM10-mediated shedding (27, 52). When the recombinant ectodomain of GPNMB (rGPNMB; 50 ng/ml) was extracellularly added to NHEKs treated with H_2_O_2_ (0.01–1.0 mM) following GPNMB knockdown, decreased viability was alleviated in NHEKs treated with low concentrations of H_2_O_2_ (0.01 and 0.1 mM) (*p* < 0.005) (Fig. 7*A*). The viability of NHEKs treated with a relatively low concentration of H_2_O_2_ (0.2 mM) was completely restored by rGPNMB in a dose-dependent manner (Fig. 7*B*); however, the viability of NHEKs treated with 1 mM H_2_O_2_ was not recovered by adding rGPNMB (Fig. 7*A*).

## Discussion

GPNMB is expressed in the basal epidermal layer of healthy skin but is lost specifically in vitiligo lesions (27). In this study, the role of GPNMB in KCs was examined and was demonstrated to have a protective role against H_2_O_2_-induced oxidative stress (Fig. 1). Notably, although the NRF2/KEEP1 system was active in MCs, it was not involved in the anti-oxidative response to H_2_O_2_ in undifferentiated NHEKs. Identical results were obtained in the NHEKs from another donor (Fig. S1). In cultured immortalized HaCaT KCs, NRF2 has been reported to be responsible for H_2_O_2_-induced oxidative stress (25, 26). Although limited information is available on primary undifferentiated NHEKs, differentiated NHEKs have been reported to express H_2_O_2_-induced NQO-1 in an NRF2-dependent manner (63). Furthermore, Durchdewald *et al.* reported that electrophilic compounds activated Nrf2 to express Nrf2 target genes in mouse KCs, whereas H_2_O_2_ had no effect, even though these treatments substantially increased intracellular ROS levels (64). This closely aligns with our findings (Fig. 2). This suggests that GPNMB is involved in the antioxidant response of undifferentiated NHEKs. In vitiligo skin, epidermal H_2_O_2_ levels are increased, while catalase and SOD levels are decreased and increased, respectively (15, 16). Therefore, reduced GPNMB expression in vitiligo lesions may play an important role in their pathophysiology, particularly in the sensitivity of KCs to oxidative stress.

**Figure 8:**
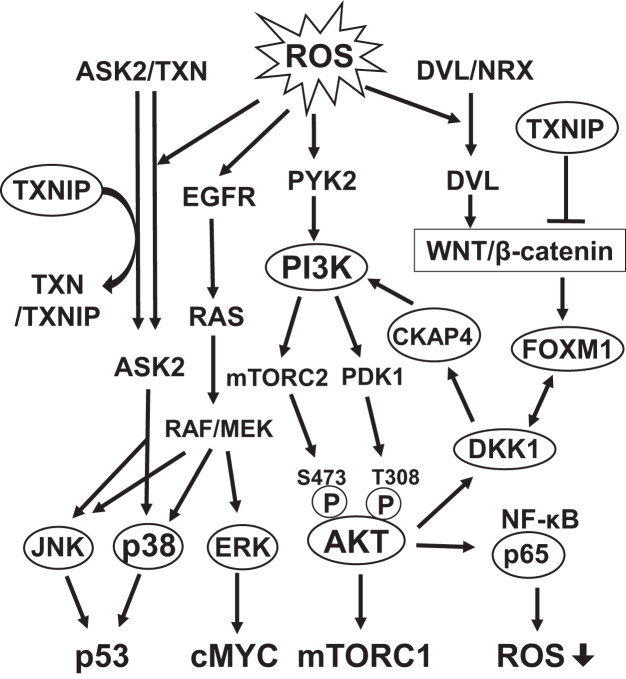
**Proposed signaling pathway activated by oxidative stress in NHEKs.** Molecules enclosed in ovals indicate those described in this study. H_2_O_2_-induced ROS stimulates an increase in intracellular Ca^2+^ following the autophosphorylation of PYK2, resulting in PI3K phosphorylation. Alternatively, DKK1 can bind to CKAP4 and activate the PI3K/AKT pathway. Thus, AKT is fully activated through phosphorylation by both PDK-1 and mTORC2, which in turn activates mTORC1 to promote survival in cells and reduces ROS levels *via* p65 phosphorylation. H_2_O_2_ activates the RAS/RAF/MEK pathway to phosphorylate ERK *via* transactivation of EGFR. ASK2 is involved in the phosphorylation of JNK and p38, which is regulated by the available amounts of TXN and TXNIP. ROS and TXNIP increase free ASK2 levels from the ASK2/TXN complex by TXN oxidation and trapping TXN in a TXN/TXNIP complex, respectively. ROS activates the WNT/β-catenin pathway by releasing free DVL from the DVL/NRX complex through NRX oxidation. Additionally, TXNIP suppresses WNT/β-catenin signaling, which increases DKK1 production *via* the β-catenin/FOXM1 pathway. EGFR, EGF receptor; DVL, disheveled segment polarity protein; NRX, nucleoredoxin; mTORC, mammalian target of rapamycin complex; PDK1, 3-phosphoinositide-dependent protein kinase-1; PYK2, proline-rich tyrosine kinase 2.

H_2_O_2_-induced oxidative stress in NHEKs (80% survival) triggered typical responses, including increased phosphorylation of PI3K, AKT, ERK, p38, JNK, and p65 (Figs. 3 and 8), which determine cell death or survival. GPNMB knockdown reduced the AKT^T308^ and AKT^S473^ phosphorylation levels 1 h after H_2_O_2_ treatment, although PI3K phosphorylation was undetectable due to its transient phosphorylation 30 min after H_2_O_2_ treatment (Figs. 3 and 4). The reduced t-AKT, t-PI3K, and t-JNK expression levels following their phosphorylation might be due to specific H_2_O_2_-induced oxidative stress responses, but further studies are needed (Figs. 3 and S3). These results suggested that the PI3K/AKT axis was downregulated by decreased GPNMB expression, resulting in reduced mTORC1-dependent KC survival (Fig. 8) (65, 66).

Findings that GPNMB knockdown reduced DKK1 production suggested the involvement of WNT/β-catenin signaling in suppressing the PI3K/AKT axis (Fig. 5, *B* and *C*) (59, 67). DKK1 activates PI3K/AKT *via* CKAP4, which is followed by FOXM1-dependent DPP1 production (58, 59, 68). Decreased FOXM1 expression, along with reduced DKK1/CKAP4/AKT signaling, suppressed DKK1 production (Figs. 4, *A* and *B* and 5, *B*–*D*). Furthermore, decreased FOXM1 expression suggested that DKK1 production *via* the WNT/β-catenin/FOXM1 axis was suppressed (Fig. 8).

H_2_O_2_-induced ROS production accelerated JNK and p38 phosphorylation (Fig. 3), possibly by releasing free ASK2 release from the inert ASK2/TXN complex (Fig. 8) (60, 61). The GPNMB knockdown-induced decrease in *thioredoxin reductase 1 (TXNRD1)* mRNA expression (Fig. 6*A*), which regenerates the reduced form of TXN, may promote an increase in free ASK2 by decreasing ASK2/TXN formation (Fig. 8). A significant increase in TXNIP production following GPNMB knockdown (Fig. 6, *A* and *B*) may also increase free ASK2 levels by forming a TXN/TXNIP complex with ASK2/TXN (Fig. 8) (62). Further quantitative measurement of phosphorylated ASK2/total ASK2 levels is required for the involvement of the ASK2/TXN/TXNIP pathway in decreased KC GPNMB expression and vitiligo pathogenesis. TXNIP has been reported to suppress WNT/β-catenin signaling (69, 70), suggesting that increased TXNIP is involved in reducing DKK1 production *via* the WNT/β-catenin/FOXM1 axis (Fig. 8).

Taken together, these results suggest that the decreased expression of KC GPNMB in vitiligo lesions triggers increased sensitivity to H_2_O_2_-induced oxidative stress and decreased signaling in the WNT/β-catenin axis. This is consistent with previous reports that vitiligo lesions exhibit basal vacuolization, KC ballooning, spongiosis, and an increase in the number of apoptotic caspase-3-positive cells (9). Furthermore, there has recently been increasing interest in the WNT/β-catenin signaling pathway, which is downregulated in vitiligo MCs (71, 72). The significant decrease in DKK1 production by GPNMB knockdown suggests that GPNMB is a key molecule regulating the WNT/β-catenin signaling pathway in vitiligo KCs.

Our previous study showed that GPNMB expressed in NHEKs is shed by ADAM10 to release sGPNMB (27), which then exerts multiple receptor-dependent functions. In this study, extracellular sGPNMB alleviated H_2_O_2_-mediated NHEK cytotoxicity, which was enhanced by GPNMB knockdown (Fig. 7), suggesting that sGPNMB is involved in cellular responses to oxidative stress. Conversely, sGPNMB protects melanocytes from oxidative stress by inhibiting AKT phosphorylation (56). Thus, the actions of sGPNMB may differ depending on which receptors and signaling pathways are used. For example, sGPNMB can bind to various receptors, including Na^+^/K^+^-ATPase (46, 54), CD44 (31, 44), and integrins (40). sGPNMB-mediated neuroprotection in Schwann cells and progression of brain blastoma has been reported through AKT and MEK/ERK pathways *via* binding to the α-subunit of Na^+^/K^+^-ATPase (47, 54), while, the receptors utilized by sGPNMB in HHEKs require further investigation. Furthermore, in the current study, the effect of sGPNMB on NHEK cytotoxicity was limited to treatment with low H_2_O_2_ concentrations (<0.2 mM) (Fig. 7*B*). The intracellular domain of membrane-bound GPNMB may also be involved in the response of the NHEKs to H_2_O_2_-mediated oxidative stress; however, further studies are needed to elucidate this possibility. Moreover, this study is limited owing to the use of *in vitro* experiments with cultured NHEKs from limited donors; further *in vitro* validation and studies on vitiligo skin are warranted.

Vitiligo skin is characterized by the specific loss of MCs, which is caused by IFN-γ-producing CD8^+^T cells targeting melanocyte-specific antigens. Mechanisms independent of acquired immunity have been proposed, including the formation of floating MCs owing to weakened interactions between basement membranes and MCs, as well as between KCs and MCs (12, 17, 18). Additionally, T cells (5, 6), KCs (18), and mast cells (14) have been reported to produce IFN-γ, which downregulates GPNMB (27), and subsequently, the decreased GPNMB levels may disrupt the KC-MC interaction *via* integrin-β1 (40). Therefore, IFN-γ-mediated decrease in KC GPNMB expression may partially explain the pathogenesis of vitiligo.

In summary, this study revealed that GPNMB protects KCs from H_2_O_2_-induced oxidative stress through enhanced PI3K/AKT signaling and suppressed JNK and p38 phosphorylation. Furthermore, reduced GPNMB suppressed the WNT/β-catenin signaling pathway in NHEKs, as indicated by reduced DKK1 and increased TXNIP production. These findings are consistent with the pathological features of vitiligo KCs and may explain the decreased cancer risk of vitiligo KCs (73) because WNT/β-catenin signaling is downregulated (58, 59, 67, 68, 70, 71) and KCs damaged by oxidative stress are easily cleared from vitiligo lesions. These findings highlight the multifaceted functions of GPNMB in the pathogenesis of vitiligo and decreased cancer risk, expand our understanding of vitiligo pathogenesis, and propose new targets for treatment.

## Experimental procedures

### Cell culture and stimulation

NHEKs were purchased from Lifeline Cell Technology and maintained in MCDB 153 HAA medium (Peptide Institute) with 0.07 mM Ca^2+^, 5 mg L^−1^ insulin, 100 ng L^−1^ epidermal growth factor, 180 μg L^−1^ hydrocortisone, 6.1 mg L^−1^ monoethanolamine, 14.1 mg L^−1^ *O*-phosphorylethanolamine, and 0.4% (v/v) bovine pituitary. Immortalized human melanocytes-hTERT (hTERT-melanocytes, T0462; Applied Biological Materials) were cultured according to the manufacturer’s protocol. PriGrow II medium (TM002; Applied Biological Materials) with 10% fetal bovine serum (FBS) was used. The cells were cultured in a humidified atmosphere with 5% CO_2_ at 37 °C.

Gene knockdown was performed using 10 nM target siRNAs and the HiPerFect transfection reagent (QIAGEN). The medium was replaced after 24 h of transfection. The siRNA oligonucleotide sequences were *GPNMB* siRNA:5′- GGAGCUGAGUAGGAUUCCUGAUGAA-3′, *NRF2* siRNA:5′-CAAACUGACAGAAGUUGACAAUUAU-3′ and nontarget control (NT) siRNA (Thermo Fisher Scientific).

When necessary, NHEKs were stimulated with H_2_O_2_ (FUJIFILM Wako Pure Chemical), t-butylhydroquinone (tBHQ, Tokyo Chemical Industries), or pretreated with MG-132 (FUJIFILM Wako Pure Chemical). Human rGPNMB (Lys23 - Asn486) was purchased from R&D systems (2550-AC).

### qRT-PCR

Total RNA extraction was performed using a NucleoSpin RNA kit (Takara Bio). Complementary DNA was prepared at 37 °C for 15 min using PrimeScript RT Master Mix enzyme (Takara Bio). PCR amplification of TB Green*Premix Ex Taq*II (Takara Bio) was conducted; 45 cycles were performed under the following conditions: denaturation for 5 s at 95 °C and annealing and extension for 30 s at 60 °C, using CFX96 Deep Well Real-Time system (Bio-Rad Laboratories). *ATP5F1* was used as the reference gene for normalization. The PCR primer sequences are shown in Table S1.

### Immunoblotting analysis

NHEK cell lysates were prepared using RIPA buffer (FUJIFILM Wako Pure Chemical) containing a phosphatase inhibitor cocktail (Nacalai Tesque). Protein concentrations were measured using the DC Protein Assay Kit (Bio-Rad Laboratories). The samples were separated on SuperSepAce gels (FUJIFILM Wako Pure Chemical) and transferred to PVDF membranes (Merck, Darmstadt, Germany). After blocking at room temperature (15–25 °C) for 30 min with Blocking OneP (Nakarai Tesque), the membrane was incubated overnight with specific antibodies at 4 °C. The primary antibodies used are listed in the (Table S2).

Primary antibodies were detected using horseradish peroxidase-conjugated secondary antibodies (GE Healthcare). Specific bands were detected using ImmunoStar LD (FUJIFILM Wako Pure Chemical) and an Amersham Imager 680 (GE Healthcare). Expression levels were determined by quantifying the intensity using Amersham Imager 680 Analysis software (GE Healthcare).

### Cell viability assay

Cells were seeded into 96-well plates. After reaching confluence, the cells were stimulated under varying conditions. For cytotoxicity assay, Cell Counting Kit-8 (CCK-8; Dojindo) was used. CCK-8 solution, diluted one-fourth with culture medium, was added 20 μl/well and incubated in a CO_2_ incubator set at 37 °C for 1 h. Absorbance was measured at 450 nm using an EPOCH optical density reader (BioTek Instruments). The absorbance at 660 nm was also measured as a background value.

### Next-generation sequencing

Total RNA was isolated from NHEKs using the RNeasy mini kit (QIAGEN) and verified to have an RNA integrity number (RIN) greater than 7.0. From 100 ng total RNA, poly(A) RNA was isolated using the NEBNext poly(A) mRNA magnetic isolation module (NEB). The RNA library was then prepared according to the NEBNext Ultra II RNA Library Prep Kit protocol. Briefly, the RNA was reverse-transcribed to cDNA and amplified by PCR. A barcode sequence was inserted into the PCR product to label the sample and sequenced on a NextSeq500 (Illumina. Inc) using a 75 bp paired-end sequencing kit. The detected gene reads were mapped to a *Homo sapiens* GRCh37 reference sequence after trimming using CLC Genomics Workbench (ver 12.0.3), and the mapping ratio was greater than 97%. Tag counts were calculated, and reads for each gene and transcript I.D. were counted using the RNA-Seq analysis tool (ver 2.18, CLC Genomics Workbench). Values were normalized to transcripts per million (TPM) based on gene length and read count (74, 75). Relative expression level (determined as TPM of target gene *versus* TPM of ATP5F1) was expressed as the mean ± standard error of the mean (S.E.M.)

### Statistical analysis

For the statistical analysis of RT-qPCR results, immunoblotting assays, and next-generation sequencing, the mean ± standard error of the mean (S.E.M.) was calculated for normalized distributions from different culture wells. Comparisons between multiple variables were calculated using Tukey's test and Student's *t* test (Figs. 1, *A* and *D* and 5*C*). Statistical analysis was performed using JMP Pro software (ver. 17.2, JMP Statistical Discovery). *p* < 0.05 was considered statistically significant. The varying statistical significance is indicated by: ∗*p* < 0.05; ∗∗*p* < 0.01; ∗∗∗*p* < 0.001.

## Data availability

The data that support the findings of this study are available from the corresponding author [inoshin@gifu-pu.ac.jp] upon reasonable request.

## Supporting information

This article contains supporting information.

## Conflict of interest

The authors declare that they have no conflicts of interest with the contents of this article.
