# Supplementary material for: The glycoprotein GPNMB protects against oxidative stress through enhanced PI3K/AKT signaling in epidermal keratinocytes
Source: J Biol Chem. 2025 Feb 11;301(3):108299. doi: 10.1016/j.jbc.2025.108299 (PMC11930081; doi:10.1016/j.jbc.2025.108299)
Supplement: Supplementary tables [file mmc1.docx]

**Supplementary Table 1.** Primers used in this study.

| Target | Direction | Sequence |
| --- | --- | --- |
| *GPNMB* | Forward | 5’- TCCAGATGACAGACGTCCTGATG -3’ |
| *GPNMB* | Reverse | 5’ - TCTGGGTGATCTCGCAGGTG -3’ |
| *NRF2* | Forward | 5’- TGGGCCCATTGATGTTTCTG -3’ |
| *NRF2* | Reverse | 5’- TGCCACACTGGGACTTGTGTTTA -3’ |
| *HO-1* | Forward | 5’- TTGCCAGTGCCACCAAGTTC -3’ |
| *HO-1* | Reverse | 5’- TCAGCAGCTCCTGCAACTCC -3’ |
| *GCLC* | Forward | 5’- GCATTATTGACGAACTGGCTACA -3’ |
| *GCLC* | Reverse | 5’- CTTAATCAATTTCTGGCTCACTGG -3’ |
| *NQO-1* | Forward | 5’- GTGGCAGTGGCTCCATGTACTC -3’ |
| *NQO-1* | Reverse | 5’- GAGTGTGCCCAATGCTATATGTCAG -3’ |
| *ATP5F1* | Forward | 5’- GAAGCAGGCTTCCATCCAACA -3’ |
| *ATP5F1* | Reverse | 5’- TCGTTCCCGGTAAGTAACTTCCAA -3’ |

**Supplementary Table 2.** Antibodies used in this study.

| Antibody |  | Primary  / Secondary | Dilution | Manifacture |
| --- | --- | --- | --- | --- |
| Anti-GPNMB #AF2550 |  | Primary | 1:500 | R&D systems, MN, USA |
| Anti-GAPDH #32233 |  | Primary | 1:2000 | Santa Sruz Biotechnology, TX, USA |
| Anti-NRF2 #13032 |  | Primary | 1:1000 | Santa Cruz Biotechnology, TX, USA |
| Anti-HO-1 #ADI-SPA-895 |  | Primary | 1:1000 | Enzo Life Sciences, Burlington, Canada |
| Anti-NQO1 #3187 |  | Primary | 1:1000 | Cell Signaling Technology, MA, USA |
| Anti-p-AKT (S473) #4058 |  | Primary | 1:1000 | Cell Signaling Technology, MA, USA |
| Anti-p-AKT (T308) #9275 |  | Primary | 1:1000 | Cell Signaling Technology, MA, USA |
| Anti-AKT #9272 |  | Primary | 1:1000 | Cell Signaling Technology, MA, USA |
| Anti-p-PI3K #4228 |  | Primary | 1:1000 | Cell Signaling Technology, MA, USA |
| Anti-PI3K #4257 |  | Primary | 1:1000 | Cell Signaling Technology, MA, USA |
| Anti-p-p65 #3033 |  | Primary | 1:1000 | Cell Signaling Technology, MA, USA |
| Anti-p65 #8242 |  | Primary | 1:1000 | Cell Signaling Technology, MA, USA |
| Anti-p-Erk #4370 |  | Primary | 1:1000 | Cell Signaling Technology, MA, USA |
| Anti-Erk #4695 |  | Primary | 1:1000 | Cell Signaling Technology, MA, USA |
| Anti-p-p38 #4511 |  | Primary | 1:1000 | Cell Signaling Technology, MA, USA |
| Anti-p38 #8690 |  | Primary | 1:1000 | Cell Signaling Technology, MA, USA |
| Anti-p-JNK #4668 |  | Primary | 1:1000 | Cell Signaling Technology, MA, USA |
| Anti-JNK #9252 |  | Primary | 1:1000 | Cell Signaling Technology, MA, USA |
| Anti-Dkk1 #48367 |  | Primary | 1:500 | Cell Signaling Technology, MA, USA |
| Anti-TXNIP #18243-1-AP |  | Primary | 1:500 | Proteintech, IL, USA |
| Anti-Goat IgG #NA9340V |  | Secondary | 1:10000 | GE Healthcare Bioscience, NJ, USA |
| Anti-Mouse IgG #7076 |  | Secondary | 1:10000 | Cell Signaling Technology, MA, USA |
| Anti-Rabbit IgG #A27014 |  | Secondary | 1:10000 | Invitrogen, CA, USA |
