# Supplementary material for: The glycoprotein GPNMB protects against oxidative stress through enhanced PI3K/AKT signaling in epidermal keratinocytes
Source: J Biol Chem. 2025 Feb 11;301(3):108299. doi: 10.1016/j.jbc.2025.108299 (PMC11930081; doi:10.1016/j.jbc.2025.108299)
Supplement: Supplemental Figures [file mmc2.pdf]

Fig. S1.

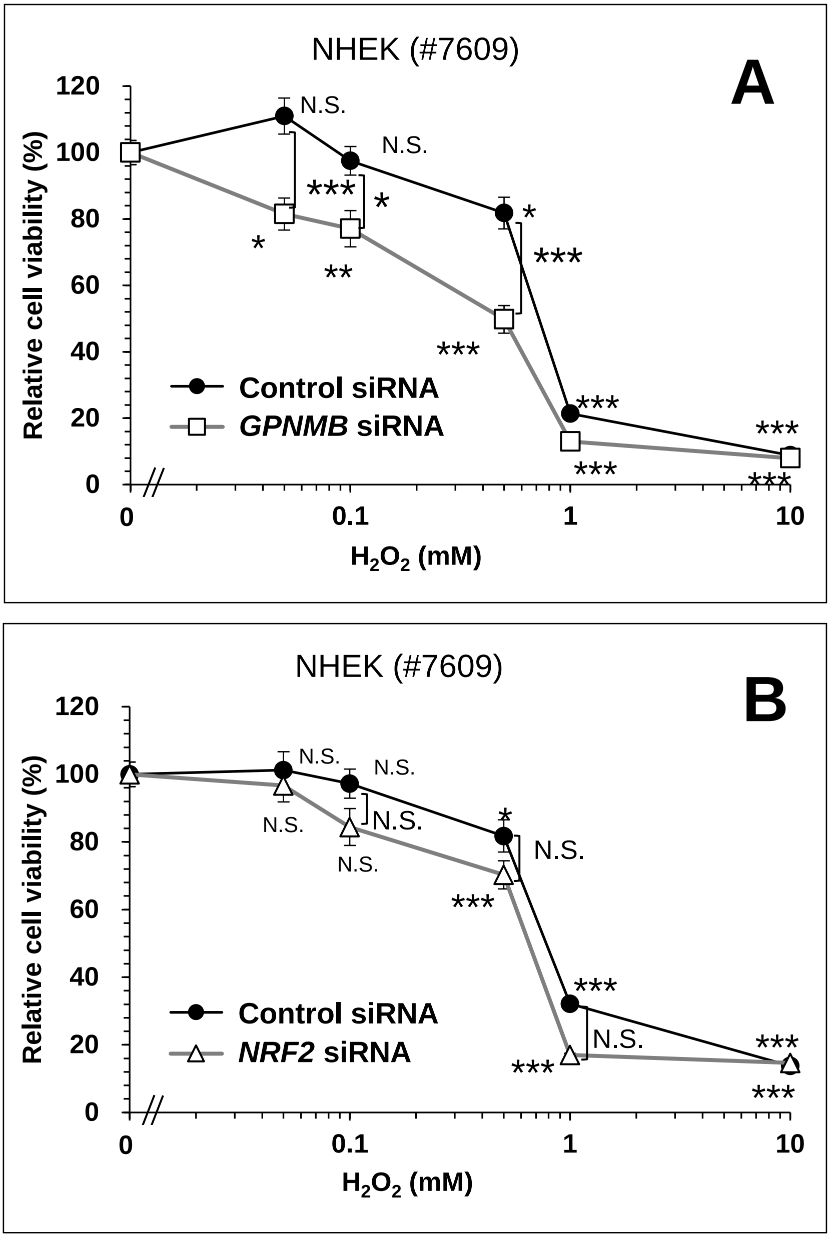

Figure S1. Knocking down *GPNMB*, but not *NRF2*, increases H_2O_2 -induced cytotoxicity in normal human epidermal keratinocytes (NHEKs).

Another donor NHEKs (Lot. 7609) were used for Fig. S1. (A, B) After transfection of the control (closed circle), *GPNMB* (open square, A), or *NRF2* (open triangle, B) siRNA for 48 h, NHEKs were stimulated with H_2O_2 (0.03-10 mM) for 24 h. The cytotoxicity of H_2O_2 was detected using Cell Counting Kit-8. Values represent the mean ± S.E.M. (n = 8). * $p < 0.05$, ** $p < 0.01$, *** $p < 0.001$ (Tukey's test). N.S.: not significant.

Fig. S2.

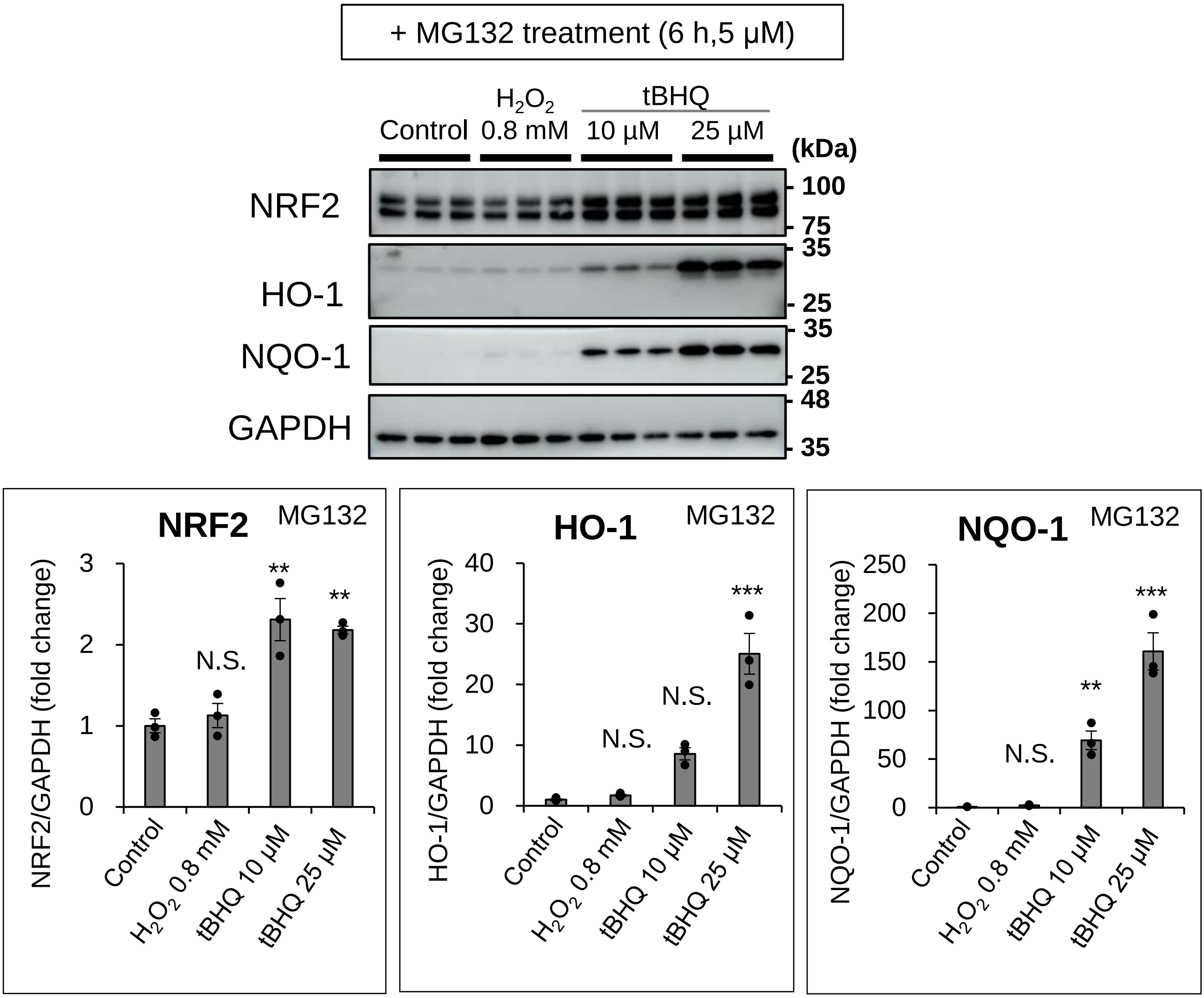

Figure S2. NRF2 signaling does not play a role in H_2O_2 -induced oxidative stress in NHEKs. NHEKs were pretreated with the proteasome inhibitor MG132 (5 μM) for 6 h to stabilize the NRF2-KEAP1 complex. The expression of NRF2, HO-1, and NQO-1 proteins was detected by immunoblotting following treatment with or without H_2O_2 (0.8 mM) and *t*BHQ (10 or 25 μM) for 24 h. GAPDH was used as a loading control. Under these conditions, NRF2 protein was detected in all lanes. The relative band intensity was calculated, and the fold change was calculated as a ratio. The non-treated control was set as 1.0. Values represent the mean ± S.E.M. (n = 3). * $p < 0.05$, ** $p < 0.01$, *** $p < 0.001$ versus non-treated control (Tukey's test). N.S.: not significant.

Fig. S3

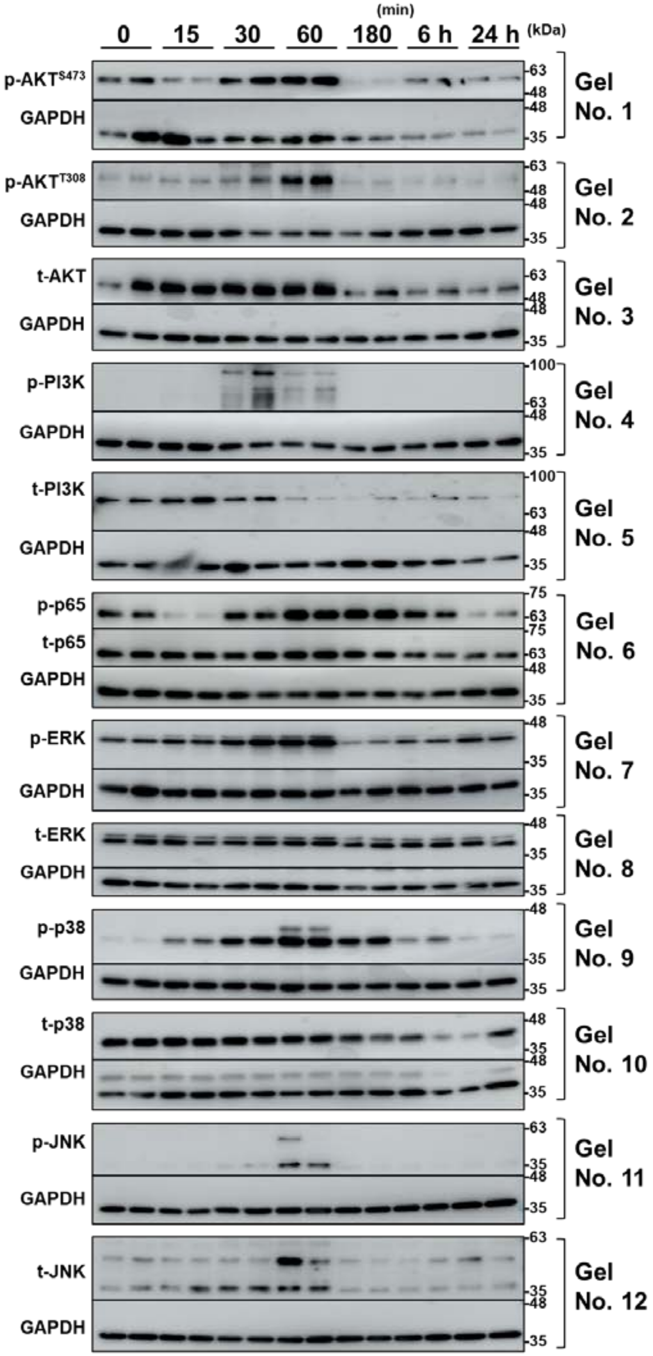

Figure S3. All GAPDH expression levels during the time course of the experiment presented in Fig. 3.

NHEK cells were treated with 0.8 mM of H_2O_2 for 15 min to 24 h. Each target protein was analyzed by immunoblotting. The same samples were loaded onto all gels, with GAPDH used as a loading control for all twelve gels.
